# Stabilizing lithium metal anode by octaphenyl polyoxyethylene-lithium complexation

**DOI:** 10.1038/s41467-020-14505-8

**Published:** 2020-01-31

**Authors:** Hongliu Dai, Xingxing Gu, Jing Dong, Chao Wang, Chao Lai, Shuhui Sun

**Affiliations:** 1 School of Chemistry and Materials Science, Jiangsu Normal University, 221116 Xuzhou, Jiangsu China; 2 Institut National de la Recherche Scientifique, Center for Energy, Materials and Telecommunications, Varennes, QC J3X 1S2 Canada; 3 College of Environment and Resources, Chongqing Technology and Business University, 400067 Chongqing, China

**Keywords:** Chemistry, Energy science and technology

## Abstract

Lithium metal is an ideal anode for lithium batteries due to its low electrochemical potential and high theoretical capacity. However, safety issues arising from lithium dendrite growth have significantly reduced the practical applicability of lithium metal batteries. Here, we report the addition of octaphenyl polyoxyethylene as an electrolyte additive to enable a stable complex layer on the surface of the lithium anode. This surface layer not only promotes uniform lithium deposition, but also facilitates the formation of a robust solid-electrolyte interface film comprising cross-linked polymer. As a result, lithium|lithium symmetric cells constructed using the octaphenyl polyoxyethylene additive exhibit excellent cycling stability over 400 cycles at 1 mA cm^−2^, and outstanding rate performance up to 4 mA cm^−2^. Full cells assembled with a LiFePO_4_ cathode exhibit high rate capability and impressive cyclability, with capacity decay of only 0.023% per cycle.

## Abstract (web summary)

Despite the large theoretical promise of Li metal anode, the dendrite growth poses a serious safety challenge. Here the authors address this issue by adding octaphenyl polyoxyethylene as an electrolyte additive which facilitates the formation of a dual-functional layer and excellent performance.

## Introduction

The increasing high demand of chargeable portable devices, electric vehicles, and large-scale grid energy storage has prompted intensive research into highly energy-dense lithium-based batteries^1^. In particular, batteries combining the highest-capacity anode (lithium metal) with high-capacity cathodes (including oxygen and sulfur) have attracted significant attention. However, the major obstacles hindering the widespread practical applications of Li-O_2_ and Li-S batteries are the problems related to the lithium metal anode^2^. Issues resulted from dendritic lithium deposition, infinite volume change, and unstable solid-electrolyte interface (SEI) layers all contribute to the impracticality of lithium metal batteries^2–4^. Over the past several decades, researchers have developed various strategies to counteract these obstacles, including replacing Li metal with a LiX alloy^5^, developing new solid electrolytes or optimizing electrolyte components^6–9^, modifying separators^10–13^, constructing an artificial upper interfacial layer for Li metal anodes^14–18^, designing two-dimensional/three-dimensional (2-D/3-D) Li-hosting materials^4,19–21^, and various other techniques^22^.

Several studies have shown that heterogeneous deposition of lithium is the main cause of dendritic lithium growth^23^. Therefore, many approaches to improve lithium metal-based batteries are aimed at regulating the uniformity of Li-ion distribution on the surface of the anode, in order to inhibit dendritic growth during plating and stripping^24^. Recently, Wu et al. reported a functional bilayer composite separator (GO-g-PAM) in which hydrophilic polyacrylamide chains homogenize Li deposition and suppress dendrite growth^25^. Moreover, Lu et al. directly introduced a quaternized polyethylene terephthalate nonwoven fabric (q-PET) as a multifunctional interlayer to simultaneously regulate Li ion distribution and reduce anion concentration gradients by utilizing the strong Li^+^ affinity of polar ester functional groups on the backbone of q-PET^26^. The application of these artificial interfacial layers results in dendrite-free lithium metal anodes, enabling the production of a stable SEI film, which prevents continuous consumption of electrolyte. However, the introduction of an additional functional layer on the separator or anode is fiscally ineffective, and also adds further complexities to the manufacturing process. Furthermore, the aforementioned strategies are all at a macro-scale, rendering their effectiveness highly dependent on the quality of surface contact with the electrolyte. Finally, while the introduction of an additional layer increases the electrode quality, it is at the expense of the overall energy density of the battery. In lieu of these macro-scale solutions, one potential alternative relies on the optimization of electrolyte components (e.g., the introduction of additives) to achieve the same effect as these artificial surface layers. In particular, additives must both regulate lithium deposition and facilitate the construction of a robust SEI film to impede consumption of the electrolyte without hampering efficiency, in order to create a safe and long-lasting battery.

**Fig. 1:**
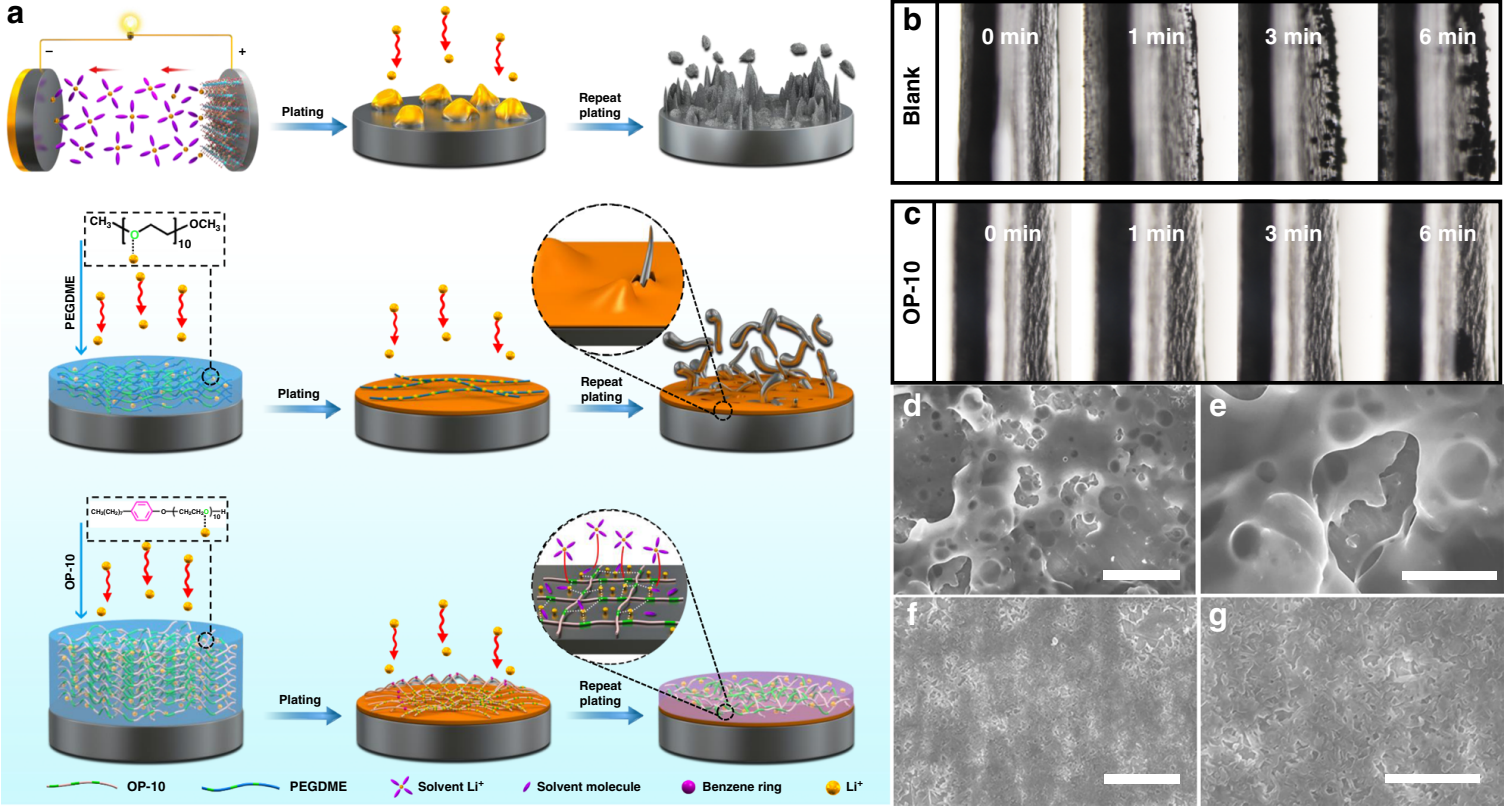
Protection mechanism of OP-10 additive and morphology characterizations of Li deposition. Schematic illustration of lithium deposition with and without various additives (**a**). In situ optical microscopy images of the Li deposition process using electrolyte without additives (**b**) and electrolyte with 5% OP-10 additive (**c**) at a current density of 4 mA cm^−2^. The thickness of the Li is about 600 µm. Top-view SEM images of lithium deposition on Cu foil at 0.5 mA cm^−2^ for 0.4 h without (**d**, **e**) and with OP-10 additives (**f**, **g**) (Scale bars: **d** 20 μm, **e** 10 μm, **f** 20 μm, **g** 10 μm).

One promising additive is polyethylene oxide (PEO), which has been thoroughly studied as a polymer electrolyte in metal lithium batteries due to its ability to coordinate lithium ions, forming a PEO/lithium complex salt (PEO/Li^+^)^27–29^. The directional motion of Li^+^ in nanochannels formed by the PEO chains can facilitate ion transport and therefore increase ionic conductivity^27^. In addition, ethylene oxide (EO) groups can react with lithium metal, forming a SEI film to improve electrolyte safety^30,31^. Based on the aforementioned qualities, the use of PEO as an electrolyte additive in lithium metal batteries to regulate Li^+^ deposition and form an ion-conductive surface layer to address the issue of lithium dendrites appears promising. However, PEO additives, such as poly(ethylene glycol) dimethyl ether (PEGDME), have been shown to produce unstable SEI layers, causing continuous consumption of the additive, significantly decreasing the batteries’ cycle life^29^. Over time, lithium dendrites form, eventually puncturing the SEI film and ultimately causing a short-circuit in the cell (Fig. 1a). This has prevented such additives from being widely investigated, despite the widespread use of PEO as a solid electrolyte^32,33^. Therefore, finding efficient polyether-based electrolyte additives that coordinate with Li^+^ to facilitate homogeneous lithium deposition while also forming a robust SEI layer is highly desirable. This will not only improve the prospects of lithium metal batteries, but also could provide theoretical guidance in the design of future electrolyte additives.

Herein, we report an effective PEO-based electrolyte additive, octaphenyl polyoxyethylene (OP-10), to regulate lithium deposition at the molecular level by constructing a stable SEI film. This allows the lithium metal anodes to remain dendrite-free even under high current densities. As shown in Fig. 1a, the OP-10 additives contain hydrophilic EO chains that coordinate with Li^+^ to form PEO/Li^+^ complexes near the anode surface, thereby controlling Li deposition. Long phenyl carbon chains form a resilient crosslinked 3-D network to improve the structural and chemical stability of the EO groups, preventing the consumption of the additives during cycling. The SEI film formed in cells containing the OP-10 additive is very flexible and stable, and successfully reduces contact between the electrolyte solvent and the anode surface, which can impede the continuous consumption of electrolyte solvent and formation of dendrites on the lithium metal anode, enabling the long-term stability of Li stripping and plating even at a high current density. By regulating lithium deposition at the molecular level, full cells comprising with a LiFePO_4_ cathode and a metal lithium anode modified with OP-10 can run up to 1000 cycles with an extremely low capacity decay of 0.023% per cycle.

## Results

### Characterization of the lithium anode surface

In situ optical microscopy was used to monitor the Li deposition process as illustrated in Fig. 1 and Supplementary Fig. 1. As shown in Fig. 1b where no additives in electrolyte, Li dendrites start to grow on the surface of the lithium foil only after 1 min. When 6 min passed, completing coating of Li dendrites on the surface of the lithium foil can be observed. In contrast, after usage of OP-10 additive in the electrolyte, there are no any lithium dendrites on lithium foil even after 6 min of plating (Fig. 1c). These results indicate that the OP-10 additives effectively inhibit the growth of dendrites on lithium foil. Similar plating experiment on copper foil was performed and the results are given in Fig. 1d–g. Without the additive, lithium deposition was observed to be highly non-uniform, resulting in the presence of several holes and large cracks on the foil surface (Fig. 1d, e). These defects can result in continuous consumption of the electrolyte and a commensurate decrease in Coulombic efficiency during cycling. In contrast, when OP-10 was added to the electrolyte, the homogeneity of lithium deposition improved markedly, with nearly imperceptible holes and cracks on the electrode surface (Fig. 1f, g).

**Fig. 2:**
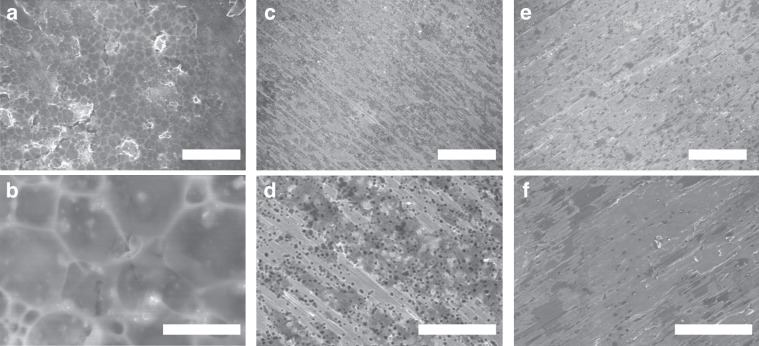
SEM analysis of the lithium anode. Top view SEM images of lithium stripping after three cycles at 0.5 mA cm^−2^ for 0.4 h with untreated electrolyte (**a**, **b**), with PEGDME (**c**, **d**), and OP-10 additives (**e**, **f**) (Scale bars: **a**, **c**, **e** 100 μm; **b**, **d**, **f** 20 μm).

A similar phenomenon is observed in the SEM analysis of copper foil surface over three cycles of Li plating and stripping (Fig. 2). Without the additive, the SEI film exhibited several defects, with holes readily observed throughout the surface (Fig. 2a, b). When a PEGDME additive was introduced into the system, the homogeneity of the SEI film was improved, but still contained many small holes (Fig. 2c, d). This non-uniform film is not stable, as it allows for a continuous reaction to happen between the Li anode and the electrolyte, eventually leading to the appearance of uncontrollable dendrite growth, as illustrated in Fig. 1a. In contrast, the addition of the OP-10 additive resulted in a dense and stable SEI film remaining after Li stripping (Fig. 2e, f). Ultimately, this stable film can prevent dendritic growth, thus improving electrochemical performance.

Meanwhile, to further verify the inhibit effect of dendrite after introducing OP-10 additives, the SEM images of Li anode and corresponding EDS element analysis after cycling are also given (Supplementary Fig. 2). With the addition of 5% OP-10, a smooth and dense surface of Li can be observed (Supplementary Fig. 2a, c), while mossy Li deposits can be observed using electrolyte with 5% PEGDME (Supplementary Fig. 2b, d). In addition, it should be noted that the content of F element in the SEI film determined by EDS elemental analysis is much higher with OP-10 additives as compared to that with PEGDME additives (Supplementary Fig. 2e, f). LiF-rich interface layers not only can make the SEI film more stable, but also can enhance Li^+^ surface diffusion rate to suppress the growth of Li dendrites during the following cycles^34^. Therefore, the Li dendrite could be effectively inhibited. It is obvious the morphology and surface composition of Li anodes are different with the addition of PEGDME and OP-10 electrolyte additives, and such differences produce the different protective effect on Li anode. As these two electrolyte additives contain the same C–O groups, thus, the protective effect difference should mainly arise from the long carbon chains.

### Electrochemical performance of symmetric cells with OP-10 additive

**Fig. 3:**
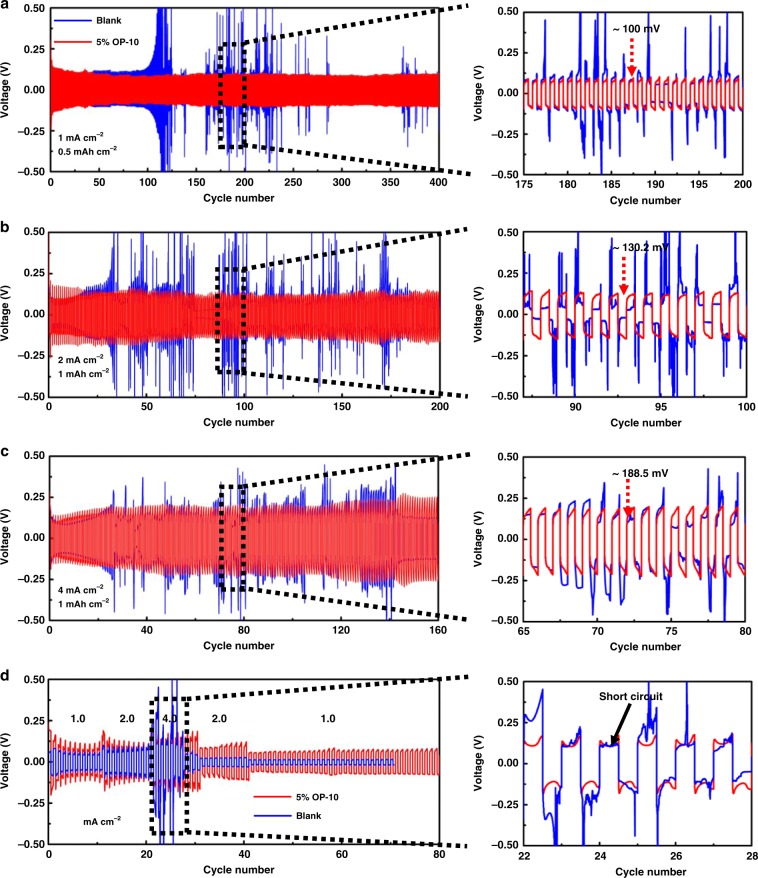
Electrochemical performance of symmetric Li|Li cells without additives (blue) and with added OP-10 (red). Cells were cycled at a current density of 1 mA cm^−2^ and a fixed capacity of 0.5 mAh cm^−2^ (**a**), a current density of 2 mA cm^−2^ and a fixed capacity of 1 mAh cm^−2^ (**b**), and a current density of 4 mA cm^−2^ and a fixed capacity of 1 mAh cm^−2^ (**c**). Rate performance of modified and unmodified electrolytes tested at current densities of 1, 2, and 4 mA cm^−2^ after 0.5 h each of Li plating and stripping (**d**).

To investigate the long cycle stability and high-rate capability of electrolytes containing OP-10, Li|Li and Li|Cu symmetric cells were analyzed (Fig. 3a–c and Supplementary Fig. 3) using electrolytes with and without additive. Cells with 5% OP-10 additive cycled stably up to 400 cycles at a current density of 1 mA cm^−2^ with a constant capacity of 0.5 mAh cm^−2^; 200 cycles at a current density of 2 mA cm^−2^ with a constant capacity of 1 mAh cm^−2^; and 160 cycles at a current density of 4 mA cm^−2^ with a unchanged capacity of 1 mAh cm^−2^. Under the same conditions, Li|Li symmetric cells without the additive showed significant fluctuations in voltage, suggesting severe instability of the lithium anode^35^. With the increasing of the cycles, the cells without additive exhibited increased polarization compared to the cells with the added OP-10. The Li anode modified by the OP-10 additive exhibited an overpotential of 100 mV at 1 mA cm^−2^, which increased slightly to 130.2 mV and 188.5 mV at 2 mA cm^−2^ and 4 mA cm^−2^, respectively (enlarged subplots of Fig. 3a–c). On the contrary, a much higher overpotential can be observed for the cells without OP-10, eventually causing a short circuit. To further illustrate the stability using OP-10 additives, the coulombic efficiency of Li|Cu symmetric cells with and without OP-10 additive were demonstrated in Supplementary Fig. 3. As shown, the cell with OP-10 additives demonstrated a stable voltage profile, indicating an excellent coulombic efficiency, while for the cell without OP-10, charge capacity decreased dramatically after 30 cycles, which indicated fast decay of coulombic efficiency. Especially, after 40 cycles, irregular voltage profiles at the trailing end can be observed for the cell without additives, indicating that Li anode has been seriously damaged^36^. The elevated polarization, increased overpotential and low coulombic efficiency in cells without additive can be attributed to the formation of lithium dendrites and pulverization of metallic lithium during repeated cycling process, which can be identified by the SEM images of anodes after cycling 50 cycles (Supplementary Fig. 4). Without the OP-10 additive, the massive dendritic and mossy Li deposits, as well as the patches of dead Li and even large cracks in the lithium foil, can be clearly observed (Supplementary Fig. 4a–c). As shown in Supplementary Fig. 4d–f, when OP-10 was added, metallic Li was stripped evenly at the surface of the modified anode and production of dendritic and dead Li was significantly decreased.

In order to further evaluate the stability of the Li anode, the rate performance of the modified and unmodified electrolytes was tested under current densities of 1, 2, and 4 mA cm^−2^ after 30 min each of Li plating and stripping (Fig. 3d). Even the voltage hysteresis was found to be more stable, and the overpotential was found to be lower for the cell without the OP-10 electrolyte additive in the initial cycles, but only after 30 cycles, the cell without the OP-10 electrolyte additive appears the short circuit phenomenon. In contrast, the cell with OP-10 electrolyte additive showed a gradually decrease overpotential and an extremely stable voltage hysteresis even after 80 cycles. To further demonstrate the advantages of OP-10 additive in lithium anode modification, the electrochemical performance comparison, including current density, areal capacity, overpotential, and cycling stability, with other works regarding electrolyte additives were listed in Supplementary Table 1. Compared with other reported organic/inorganic additives, more stable cycle performance are obtained because of the unique functions of OP-10 additives.

Finally, electrochemical impedance spectroscopy (EIS) was performed on Li|Li symmetric cells with and without the OP-10 electrolyte additives at a current density of 2 mA cm^−2^ with a constant capacity of 1 mAh cm^−2^ (Supplementary Fig. 5; Supplementary Table 2). Before cycling, only one semi-arc, associated with the charge-transfer resistance (*R*_ct_)^37^, was observed in the high-frequency range. The *R*_ct_ of the Li|Li cell with OP-10 electrolyte additives was calculated to be 156.2 Ω, slightly higher than that without additives, 140.5 Ω (Supplementary Table 2). The increased resistance in modified electrolytes is mainly because the surfactant molecules adsorbe onto the solid electrode surface and then work as a physical barrier to obstruct Li^+^ transport^37^. After cycling, two arcs associated with *R*_ct_ and *R*_SEI_ are observed (Supplementary Fig. 5). In the Li|Li cell without the OP-10 additive, the *R*_SEI_ and *R*_ct_ values increased significantly after 100 cycles due to the accumulation of dead Li and the mossy Li on the Li anode. However, in the cell containing OP-10, the *R*_ct_ and *R*_SEI_ decreased after 100 cycles (Supplementary Table 2). During cycling, a stable SEI film induced by OP-10/Li^+^ complexation was formed, which improves Li^+^ transfer and reduces the deposition of dendritic Li and mossy Li on the anode surface, thus decreasing resistance^38^.

### Regulation mechanism of OP-10 additives on the Li surface

Based on previous studies, EO units can coordinate with Li^+^ to form a PEO/Li^+^ complex^27,31^. In order to prove this combination, firstly, the ^7^Li NMR spectra of LiPF_6_ (referenced to LiCl in D_2_O) with different electrolyte systems were investigated. As shown in Supplementary Fig. 6, the chemical shift at −0.79 ppm, −0.77 ppm, and −0.66 ppm appear, which corresponds to 1 M LiPF_6_, 0.1 M PEGDME with 1 M LiPF_6_ and 0.1 M OP-10 with 1 M LiPF_6_, respectively. Both additives present obvious shift to downfield, indicating the stable complexation between both additives and Li ions^9,39^. Then the differential capacitance curves of the electrolytes with or without OP-10 additives were conducted to verify the adsorption of PEO additives onto the surface of lithium metal (Supplementary Fig. 7). The observed electrochemical stability could be due to the adsorption of the PEO-based additives onto the metal Li surface, allowing these complexes to regulate the deposition of lithium during the charge–discharge process. The capacitance of PEO-based additives was significantly lower than that of the unmodified electrolyte, and a capacitance peak appears at −0.56 V for both OP-10 and PEGDME, showing the adsorption of additive to the electrode surface and formation of a thick double layer (SEI membrane)^40,41^. This adsorption is the driving force for the increase in interfacial charge resistance, which is consistent with the EIS results described above (Supplementary Fig. 5).

**Fig. 4:**
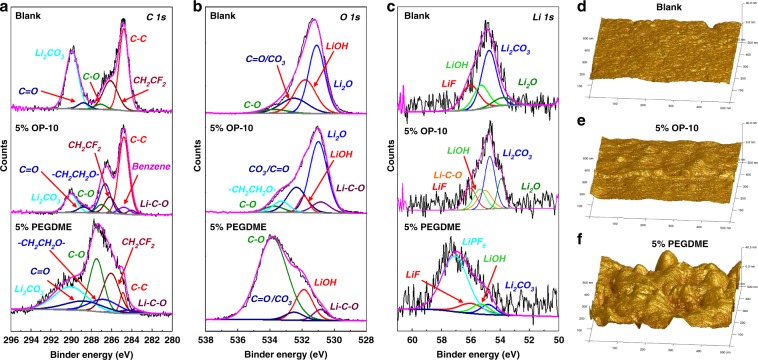
XPS and AFM analysis of Li anode in different electrolyte systems. C 1s, O 1s, and Li 1s spectra of the SEI film on the surface of the lithium anode without or with OP-10/PEGDME additives after three cycles (**a**–**c**). AFM images of the electrolyte on the copper foil: blank electrolyte (**d**), 5% OP-10 additives (**e**), 5% PEGDME additives (**f**).

In order to verify the OP-10 and PEGDME electrolyte additives were indeed involved in the SEI film formation reaction, the XPS analysis on the surface of the lithium anode without or with OP-10/PEGDME additives after three cycles were conducted. As shown in Fig. 4a–c, the C 1s, O 1s, and Li 1s spectra of a lithium foil after cycling in different electrolytes are demonstrated. In C 1s XPS spectrum (Fig. 4a), compared with untreated electrolyte, the electrolyte with OP-10 or PEGDME contains more different peaks, the visible peak at 286.7, 284.7, and 284 eV are found from the surface of Li electrode, which can be attributed to ethylene oxide group of OP-10/PEGDME, benzene group, and Li–C–O bond, respectively^42–46^. While in O 1s, the peak at 533.8 eV is assigned to the characteristic peak of Li–C–O, which is both observed in the cell using OP-10 or PEGDME as additives, but it could not observe in the cell using blank electrolyte^44^. Therefore, based on the above XPS results, it can demonstrate the OP-10 and PEGDME are involved in the film formation reaction of SEI membrane. Moreover, in Li 1s, the peak at 56 eV can be attributed to the LiF, which are consistent with the EDS results in Supplementary Fig. 2^42^.

Additionally, Fig. 4d–f shows representative AFM images from various electrolyte systems after adsorption to demonstrate different distribution morphologies on a copper surface. As shown, when the OP-10 and PEGDME additives introduced into the blank electrolyte, the EO segments can lie on the copper surface and the vesicles (raised particles in the images) are observed in Fig. 4e, f. Compared with the 5% PEGDME, the electrolyte containing 5% OP-10 has flat, smooth and uniform surface morphology, which is a favor to produce a stable interface. Moreover, the thickness of 5% OP-10 (3.5 nm) is thinner than that of 5% PEGDME (7.36 nm)^47–49^.

**Fig. 5:**
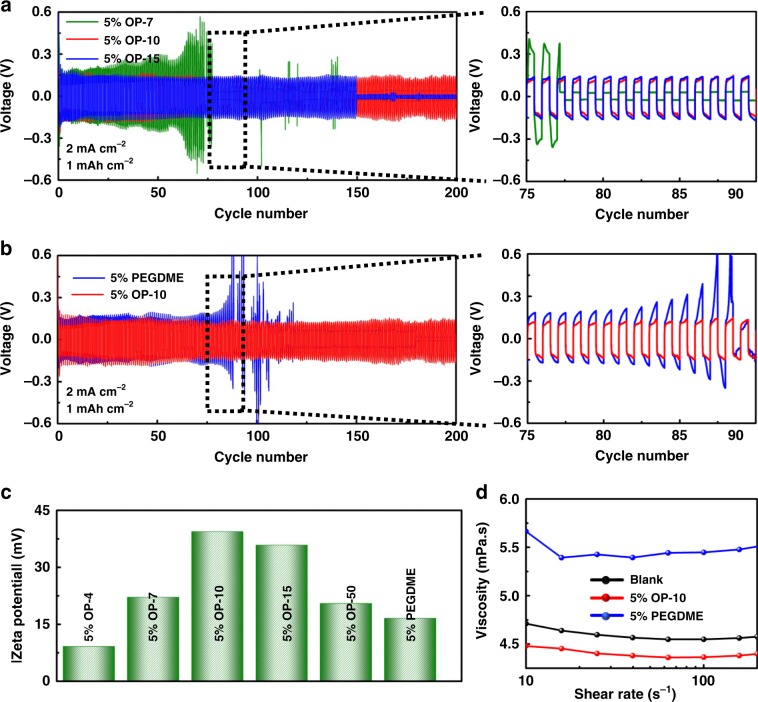
Electrochemical performance of symmetric cells and properties of different electrolytes. Cycling performance of Li|Li symmetric cells containing additives of OP-10 (red) versus OP-7 (olive), OP-15 (blue) (**a**) and PEGDME (**b**) (blue); Zeta potential of lithium with different OP additives and PEGDME additives (**c**); viscosity experiments of the electrolyte with 5% PEGDME and 5% OP-10 additives (**d**).

To clarify the effect of the EO/Li coordination in the systems containing OP-10, the cycling stability of Li|Li symmetric cells with OP-4, OP-7, OP-10, OP-15, and OP-50 electrolyte additives, containing 4, 7, 10, 15, and 50 units of EO chains, respectively, were compared (Fig. 5a, Supplementary Fig. 8a, b). From this data, it is clear that Li|Li symmetric cells containing OP-10 additives showed much higher cycling stability than those containing OP-4, OP-7, OP-15, or OP-50. This discrepancy in the stability can be attributed to the lengths of the EO chains. If the EO chains are too short, PEO/Li^+^ complexes cannot be fully formed, resulting in inadequate regulation of lithium deposition and thus, the formation of lithium dendrites^37^. However, though the increased EO chain length can promote uniform deposition of lithium, the excessively long chains may also result in unstable SEI films. In order to explain this phenomenon, the stabilities of self-assembled adsorbed layers containing EO chains of varying lengths were quantified using the Zeta potential of the lithium anode (Fig. 5c). A higher Zeta potential allows for a more stable self-assembled OP/Li^+^ layer^23^, causing the SEI film formed during cycling to be more robust. In these measurements, the lithium anode modified with OP-10 has the highest Zeta potential, as predicted by the cycling behavior.

However, the numbers of EO groups is not the only factor that influences the electrochemical performance. PEGDME possesses the same number of EO units as OP-10, but its Zeta potential value is significantly lower, which is not favorable to produce a dense SEI film as shown in Fig. 2. And therefore, as shown in Fig. 5b, its cycling behavior is much less stable. It is reasonable to assume that the improvement in the cycling behavior of cells containing OP-10 additives is mainly due to the long carbon chain of the OP-10. These chains can self-assemble and act as a template to induce the formation of the dense SEI layer responsible for the long cycling life of cells containing OP-10^37,50^. In addition, the OP-10 additives can affect the other properties of the electrolyte, which can also significantly impact cycling performance. As presented in Fig. 5d, with the same additive concentration (5%), the viscosity of OP-10 containing electrolytes is much lower than that untreated electrolyte, resulting in higher Li ion mobility. While for electrolyte with PEGDME additives, it even demonstrates a greatly higher viscosity as compared to the untreated electrolyte. According to Eq. 1:^51^

1 $$ {\it{K}} = \sum \,{\frac{{\left( {Z_i} \right)^2\,F\,C_i}}{{{\mathrm{6}}\,{\uppi}\,{r_i}}}},$$

for which *Z*_*i*_ is the charge carried by the ion (absolute value), *C*_*i*_ is the equivalent concentration, *F* is the Faraday constant, *η* is the viscosity of the solvent, *r*_*i*_ is the solvated ion radius. It is obvious the conductivity of the electrolyte is mainly decided by the concentration of Li salts and the viscosity of the solvent. For the electrolyte systems in this work, the much lower viscosity after introducing additives can offer a higher conductivity, and thus better Li ion mobility could be obtained. Such an enhancement can be directly confirmed by the testing of Li ion mobility via the pulse-field gradient method. The Li ions mobility can be increased from 7.319 × 10^−7^ cm^2^ s^−1^ to 7.386 × 10^−7^ cm^2^ s^−1^ after introducing 5% OP-10 additives. As shown in Fig. 5d and Supplementary Fig. 9, the electrolyte with 5% OP-10 additive shows the lowest viscosity, thus corresponding to better electrochemical performance. Additionally, the contact angle of the electrolyte with 5% OP-10 is only 17.67°, markedly lower than the 23.97° angle measured for the 5% PEGDME additive (Supplementary Fig. 10). This delta in contact angle indicates that the OP-10 additive is more lithiophilic, and therefore can be more easily and homogenously absorbed onto the surface of the Li anode. These advantages of OP-10, combined with the optimized chain length described above, contribute to the increased cycling stability observed in Li|Li symmetric cells containing OP-10. The lithiophilic EO chains regulate Li^+^ deposition, while the long carbon chains help to form a dense and stable SEI film to inhibit dendrite growth and change the properties of OP-10-containing electrolytes to facilitate ion transport.

Having optimized the composition and chain length of the electrolyte additive, the influence of the OP-10 concentration to the cycling stability of Li|Li symmetric cells was investigated. The cells with 5% OP-10 additive were found to exhibit the highest cycling stability and the smallest voltage polarization (Supplementary Fig. 11). When the additive concentration is below 5%, there is not sufficient OP-10/Li^+^ complexation to produce a robust layer on the anode surface. However, at excessively high additive concentrations, the intrinsic properties of the electrolyte are significantly altered so that stable OP-10/Li^+^ layer cannot be formed either. The stability of the adsorbed layer formed by the electrolytes containing different concentrations of OP-10 was quantified using the Zeta potential of the Li anode, as above. The Li foil modified by 5% OP-10 exhibits the highest Zeta potential, approximately 40 mV, corresponding to the most stable OP-10/Li^+^ layer, and therefore the most stable SEI, on the Li anode (Supplementary Fig. 12). Small amounts of OP-10 additive decrease the contact angle between the electrolyte and lithium foil (Supplementary Fig. 13), allowing the modified electrolyte to be more homogenously absorbed onto the surface of the Li anode. The decreased contact angle also corresponds to the decreased electrolyte viscosity (Supplementary Fig. 9), facilitating Li^+^ transport and thus enhancing cycling performance. However, the concentration is above 5%, the OP-10 molecules aggregate to form micelles, causing a dramatic increase in the contact angle (Supplementary Fig. 13e, f) and viscosity (Supplementary Fig. 9), hindering the ion transport and the stable SEI formation^52–56^. Due to the combination of these factors, the electrolytes with an OP-10 additive at a concentration of 5% were determined to be the most promising conditions for facilitating ion transport and reducing dendrite formation, and therefore were used for all following tests.

### Electrochemical performance of Li|LiFePO_4_ and Li|Li_4_Ti_5_O_12_ full cells

**Fig. 6:**
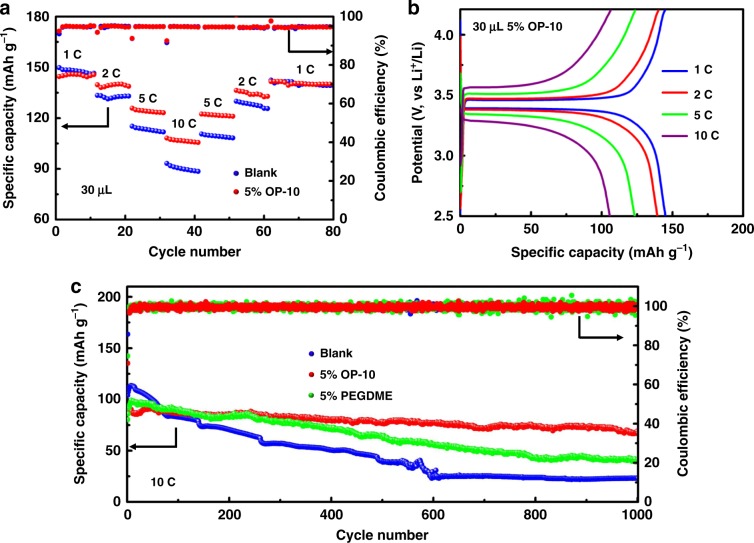
Electrochemical performance of Li|LiFePO_4_ full cells. Cycling performance at different rates (**a**), charge-discharge curves (**b**), and long-term cycling performance at 10 C (**c**) of Li|LiFePO_4_ full cells in blank or modified electrolytes.

Finally, the optimized OP-10 electrolyte was tested in Li|LiFePO_4_ and Li|Li_4_Ti_5_O_12_ full cells to determine its potential utility in practical batteries. As shown in Fig. 6a, at the lowest discharge current density of 1 C (170 mAh g^−1^), the unmodified electrolyte slightly outperformed the OP-10-modified electrolyte in the Li|LiFePO_4_ cell, but its discharge capacity decreased significantly at the higher discharge current densities ranging from 2 to 10 C. At 10 C, the LiFePO_4_ cathode in the blank electrolyte exhibited a much lower capacity of 88.5 mAh g^−1^ as compared to that of the cell with the OP-10 additive. Figure 6b and Supplementary Fig. 14 show the charge-discharge curves of the LiFePO_4_ cathode using electrolytes with and without OP-10 additives at various current densities, respectively. The charge-discharge potential plateau in the electrolyte with the OP-10 additive was relatively consistent against increasing current density, with a voltage potential gap of only 362.9 mV at 10 C (Fig. 6b). In contrast, a much higher potential gap reaching to 634.4 mV at 10 C can be observed for the full cell using the blank electrolyte (Supplementary Fig. 14).

The long-term cycling stability of Li|LiFePO_4_ cells made with blank electrolyte and with OP-10 or PEGDME additives is shown in Fig. 6c. While the cell containing blank electrolyte exhibited a slightly higher discharge capacity during the first 70 cycles, its cycling stability was the worst among the three electrolytes. After 1000 cycles, its capacity was only 23.1 mAh g^−1^, with a capacity decay as high as 0.078%. With the PEGDME additive, the cycling stability of the LiFePO_4_ cathode was improved, but the capacity dropped quickly after 300 cycles, eventually reaching 41.5 mAh g^−1^ after 1000 cycles, with a capacity decay of 0.048%. However, the cell containing the OP-10 additive was extremely stable for all cycles following the 3rd cycle, with the capacity remaining as high as 67.1 mAh g^−1^ after 1000 cycles, and exhibited a capacity decay of only 0.023%. The cycling behavior is similar in Li|Li_4_Ti_5_O_12_ full batteries, with the cell containing OP-10 showing more stable charge–discharge voltage profiles and smaller voltage gaps than that of the blank electrolyte, as well as a high reversible capacity of about 126.4 mAh g^−1^ after 1000 cycles, compared to 58.0 mAh g^−1^ for the blank electrolyte (Supplementary Fig. 15). As expected, based on Li|Li symmetric cell analysis, electrolytes containing OP-10 outperformed both blank electrolytes and PEGDME-modified electrolytes in almost all tests performed on full cells.

**Fig. 7:**
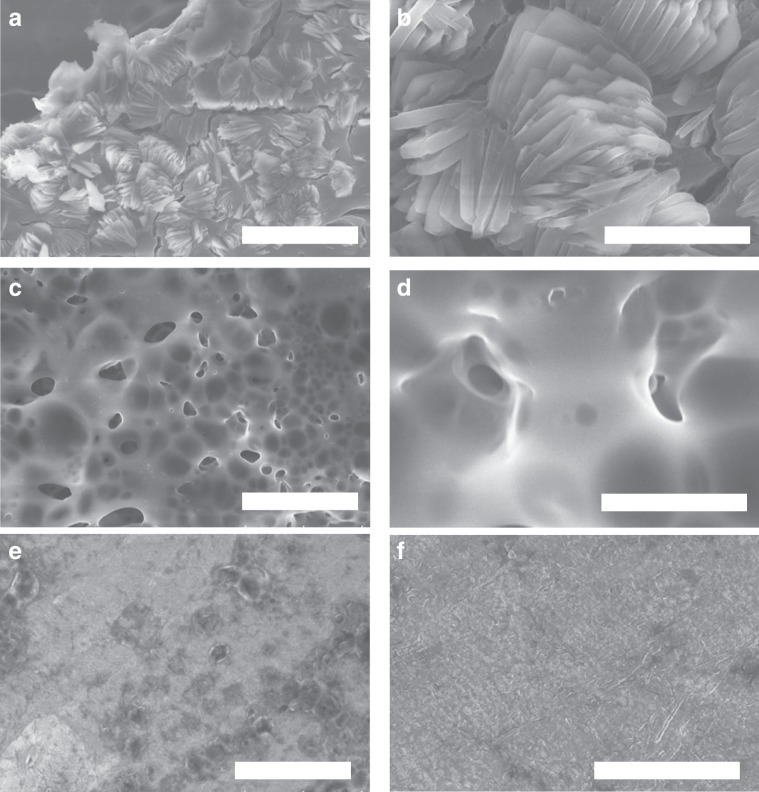
Surface morphology of Li anode after cycling. Top view SEM images of the Li anode in Li|LiFePO_4_ full cells containing blank electrolyte (**a**, **b**), and electrolytes modified with PEGDME (**c**, **d**) and OP-10 (**e**, **f**) after 1000 cycles (Scale bars: **a**, **c**, **e** 20 μm; **b**, **d**, **f** 5 μm).

To confirm the stability of the SEI layer on Li anodes in full cells containing OP-10 modified electrolytes, SEM investigations were performed after cycling. As shown in Fig. 7a, b, after 1000 charge-discharge cycles, the Li anode in the cell containing the blank electrolyte has a large number of lithium dendrites, and no obvious SEI film is observed. In cells containing the PEGDME electrolyte additive, the lithium dendrite growth was suppressed, and an SEI film can be observed on the surface of the Li anode (Fig. 7c, d). However, the SEI film is thin and unstable, which is evidenced by the appearance of vast cavities and cracks. These defects result in the consumption of the electrolyte and eventually, cell death. In contrast, when the 5% OP-10 electrolyte additive was used, not only lithium dendrite growth was completely suppressed, but a dense and stable SEI film can also be observed (Fig. 7e, f). This extremely stable cycling performance pictorially visualized in Fig. 6c can be attributed to this SEI layer.

EDS analysis was performed after cycling in order to confirm the composition of the SEI layer (Supplementary Table 3). The surface of the Li anode in the cell containing the blank electrolyte contains a higher proportion of C and O elements than that of the cell modified with OP-10, and a lower proportion F and P elements. This indicates that the formation of OP-10/Li complexes may reduce the contact between the electrolyte solvent and the lithium surface, thus reducing the involvement of solvents in the SEI formation and decreasing the C and O content of the SEI^10,57^. Meanwhile, the EO coordination with Li^+^ allows the PF_6−_ to participate in SEI film formation, resulting in a stable SEI film with more inorganic constituents, such as LiF^58^. Linear sweep voltammetry (LSV) also indicates that the stabile SEI films were formed during charge–discharge cycles in cells with OP-10 additives (Supplementary Fig. 16). No current appears for the electrolyte containing the OP-10 additive until the voltage reaches to 5 V, while current begins at 4.3 V for electrolytes containing PEGDME and 4.6 V for blank electrolytes. This indicates that the electrolyte containing the OP-10 additive is much more stable and less likely to undergo redox reactions during cycling. The combination of SEM, EDS, and LSV analyses indicates that the electrolytes containing OP-10 are more stable against redox reactions and form more stable SEI layers, thus improving the cell cycling performance.

Finally, EIS characterization was performed on Li|LiFePO_4_ cells before and after cycling (Supplementary Fig. 17a, b). Before cycling, the Li|LiFePO_4_ battery containing the blank electrolyte showed the smallest *R*_ct_ value (Supplementary Table 4), followed by the cell containing OP-10, while the cell containing the PEGDME additive had the largest *R*_ct_ (Supplementary Table 4). The modified cells exhibit higher resistance because of massive adsorption of the surfactant molecules on the surface of the solid electrode. After 500 cycles, the Li|LiFePO_4_ full cell with the blank electrolyte exhibited an even higher *R*_ct_ value than that before cycling, due to the complete destruction of its SEI layer, and the dendritic and dead lithium deposited on the electrode. Conversely, the cells with the additives form a more stable SEI film, significantly improving the Li^+^ transport and decreasing *R*_ct_ values compared to both blank electrolytes and electrolytes before cycling (Supplementary Table 4). The cell with the OP-10 additive exhibited the lowest *R*_ct_ value, corresponding to the most stable and dense SEI film, as confirmed by SEM, EDS, and LSV, which explains its excellent electrochemical performance.

## Discussion

The addition of OP-10 additives to the electrolytes results in improved homogeneity of lithium deposition due to the complexation of the lithiophilic EO functional groups with Li^+^, which tunes lithium deposition and prevents lithium dendrite growth. In addition, in contrast to the conventional PEO, the OP-10 additives not only enhance the chemical and electrochemical stability of the electrolyte, but also act as a cross-linking template to construct a stable, dense SEI layer that improves Li^+^ transport and reduces the consumption of the electrolyte. As a result, lithium anodes modified with OP-10 additives in Li|Li symmetric cells demonstrated stable cycling stability for 400 cycles at a current density of 1 mA cm^−2^. When the OP-10 modified Li anode was employed in Li|LiFePO_4_ full cells, it also exhibited excellent cycling stability and rate capability. At a high current density of 10 C, the Li|LiFePO_4_ full cells containing OP-10 exhibited a high reversible capacity of approximately 67.1 mAh g^−1^ after 1000 cycles, which is three times higher than that of the cells without additives. The electrolyte containing OP-10 was found to be extremely stable against high charge–discharge voltage, and could withstand voltages of up to 5 V. To further verify this superiority, high voltage Li|LiNi_0.8_Co_0.1_Mn_0.1_O_2_ full cells were assembled and tested. As shown in Supplementary Fig. 18b, c, the Li|LiNi_0.8_Co_0.1_Mn_0.1_O_2_ full cells with blank electrolyte, OP-10 electrolyte and PEGDME electrolyte show the similar charge–discharge voltage profiles, but after 50 cycles, this full cell with OP-10 additives shows a much more stable voltage profile than that of full cells with blank electrolyte and PEGDME electrolyte. What’s more, from the cycling performance in Supplementary Fig. 18a, Li|LiNi_0.8_Co_0.1_Mn_0.1_O_2_ full cell with OP-10 electrolyte illustrates a satisfactory reversible capacity of approximately 150 mAh g^−1^, much higher than 100 mAh g^−1^ for PEGDME electrolyte and 50 mAh g^−1^ for blank electrolyte.

To summary up, this work developed an efficient strategy to regulate the lithium deposition and construct a stable SEI film, which can significantly inhibit the dendritic lithium growth. Moreover, it also pioneers a new electrolyte additive for the production of safer, longer-lasting and higher-voltage lithium-based batteries.

## Methods

### Chemicals

Octaphenyl polyoxyethyiene (OP-4, OP-7, OP-10, OP-15, and OP-50) was obtained from Shandong Yousuo Chemical Technology Co., Ltd. Polyethylene glycol dimethyl ether (PEGDME) was received from Sigma-Aldrich Co., Ltd. LiFPO_4_ was achieved from Shanghai Darui Fine Chemical Co., Ltd. Li_4_Ti_5_O_12_ was obtained from Shenzhen Beite Rui New Energy Materials Co., Ltd.

### Materials characterization

The morphology of lithium foil before and after cycling were examined by Hitachi SU8010 scanning electron microscope (SEM), and energy dispersive spectrum (EDS) analysis were also conducted by this SEM machine. In situ monitoring of lithium dendrite formation was observed via optical microscopy (Nikon SMZ1270). The viscosity test of electrolyte was conducted by a TA-DHR2 rheometer. The wettability of ester-based electrolyte with different concentrations additives on bare lithium foil electrode were demonstrated using an automatic contact angle measuring instrument (JC2000D3M). The zeta-potential of 1.5 mg mL^−1^ lithium powder dispersions in LiPF_6_-PC/EC/DEC electrolyte was tested using a Zetasizer Nano ZS (90Plus PALS, UK). Using Bruker Avance NEO 600 MHz nuclear magnetic resonance (NMR) Spectrometer to conduct the experiment of ^7^Li NMR spectra of 1 M LiPF_6_-THF with different electrolyte systems. X-ray photoelectron spectroscopy (XPS) experiments were conducted with a monochromatized 1486.6 eV Al K Alpha radiation. Atomic force microscope (AFM) experiments were conducted by the machine of Bruker Dimension Icon. The testing of Li ion mobility was conducted by fitting the ln*I/I*_0_ – *G*^2^ plot depend on Eq. 2:^39^

2 $$ I\,{\mathrm{ = }}\,I_0\,{\mathrm{exp}}\,\left[ { - \left( {{\upgamma }}\,{\mathrm{G}}\,{\updelta} \right)^2\,D\left( {\Delta - \delta /3} \right)} \right], <Source Format="MATHML"><math><mi>I</mi><mspace width="0.25em"/><mi mathvariant="normal">=</mi><mspace width="0.25em"/><msub><mrow><mi>I</mi></mrow><mrow><mn>0</mn></mrow></msub><mspace width="0.25em"/><mi mathvariant="normal">exp</mi><mspace width="0.25em"/><mfenced separators="" open="[" close="]"><mrow><mo>-</mo><msup><mrow><mfenced separators="" open="(" close=")"><mrow><mi mathvariant="normal">{\gamma}</mi><mspace width="0.25em"/><mi mathvariant="normal">G</mi><mspace width="0.25em"/><mi mathvariant="normal">δ</mi></mrow></mfenced></mrow><mrow><mn>2</mn></mrow></msup><mspace width="0.25em"/><mi>D</mi><mfenced separators="" open="(" close=")"><mrow><mi mathvariant="normal">Δ</mi><mo>-</mo><mi>δ</mi><mo>∕</mo><mn>3</mn></mrow></mfenced></mrow></mfenced><mo>,</mo></math>$$

where *I* is the observed peak intensity, *I*_0_ is the peak intensity without gradient, *γ* is the gyromagnetic ratio of the nuclei, *G* is the applied gradient strength, *D* is the diffusion co-efficient, *δ* is the gradient length, and *∆* is the diffusion delay.

### Electrochemical measurements

All electrochemical measurements were investigated via coin cells at room temperature. All the batteries were measured in the ester-based electrolyte (30 µL). The balnk electrolytewas 1.0 M lithium hexafluorophosphate dissolved in mixing solvents of propylene carbonate (PC), ethyl carbonate (EC), and diethyl carbonate (DEC) with a volume ratio of 1:4:5. For the modifited electrolyte, 5.0% (volume) additives were added in the blank electrolyte. The Li|Li symmetrical cells were conducted at current densities of 1.0, 2.0, and 4.0 mA cm^−2^, respectively. To test the electrochemical performances of Li|LiFPO_4_ (LFP, Shanghai Darui Fine Chemical Co., Ltd.), Li|LiNi_0.8_Co_0.1_Mn_0.1_O_2_ (NCM) and Li|Li_4_Ti_5_O_12_ (LTO, Shenzhen Beite Rui New Energy Materials Co., Ltd.) batteries, LFP, NCM, or LTO were used as the cathode and Li foil was used as the anode. To fabricate the LFP, NCM, or LTO electrode, LFP, NCM, or LTO, carbon black, and polyvinylidene difluoride in a weight ratio of 7:2:1 in N-methyl-2-pyrrolidone to form a homogeneous slurry, and then coated this slurry onto the aluminum foil or copper foil. After dying at 60 °C for 12 h, all the above batteries were galvanostatically cycled on the battery testing system (LAND, CT2001). Li|LFP cells were performed between 2.5 and 4.2 V (vs. Li^+^/Li) at current densities of 1, 2, 5, and 10 C (1 C = 170 mA g^−1^). Li|LTO cells were performed between 1.0 and 2.5 V (vs Li^+^/Li) at current densities of 1, 2, 5, and 10 C (1 C = 175 mA g^−1^). Li|NCM cells were performed between 3.0 and 4.7 V (vs. Li^+^/Li) at current densities of 1 C (1 C = 280 mA g^−1^). The mass loading of the LFP, LTO and NCM electrodes are about 1.0–1.2, 1.2, and 1.5 mg cm^−2^, respectively. Linear sweep voltammetry (LSV) measurements were obtained with copper foil as the working electrode and Li foil as a counter electrode between 2.8 and 5.0 V under a scan rate of 10 mV s^−1^. Electrochemical impedance spectra (EIS) were measured using an Solartron 1287 electrochemical workstation at an amplitude of 5 mV over a frequency range between 10 mHz and 100 kHz. With the same instrument, the differential capacitance-potential curves were studied.

## Supplementary information

Supplementary Information

## Data Availability

The data that support the findings of this study are available from the corresponding author upon request.
