# Supplementary material for: Stabilizing lithium metal anode by octaphenyl polyoxyethylene-lithium complexation
Source: Nat Commun. 2020 Jan 31;11:643. doi: 10.1038/s41467-020-14505-8 (PMC6994683; doi:10.1038/s41467-020-14505-8)
Supplement: Supplementary file 1 — Supplementary Information [file 41467_2020_14505_MOESM1_ESM.pdf]

Supplementary Information

Stabilizing lithium metal anode by octaphenyl polyoxyethylene complexation

Dai et al.

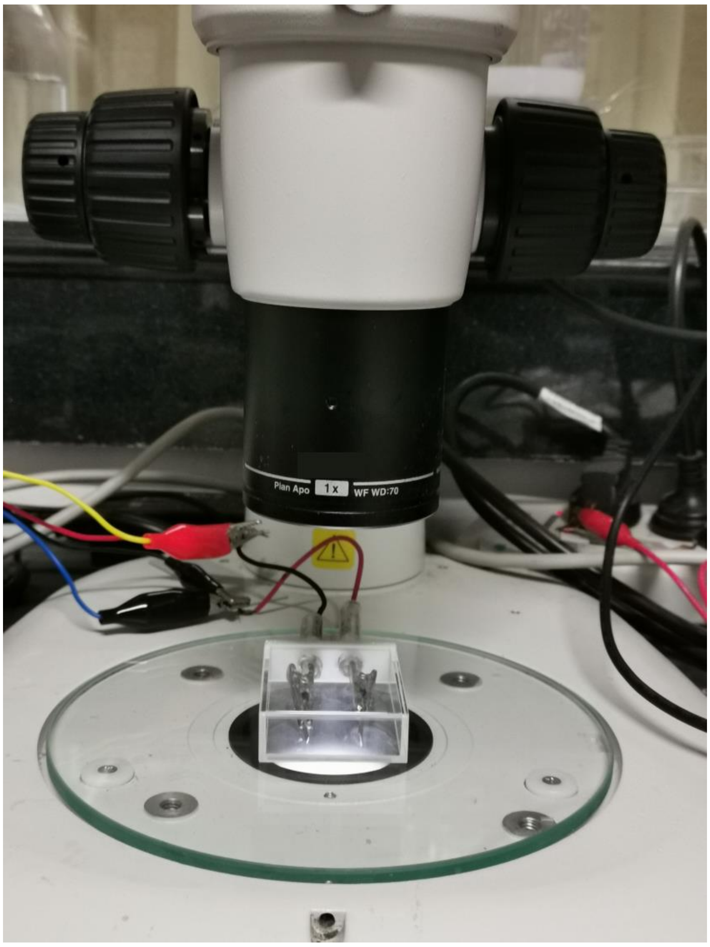

Supplementary Figure 1. Digital photo of *in situ* optical microscopy.

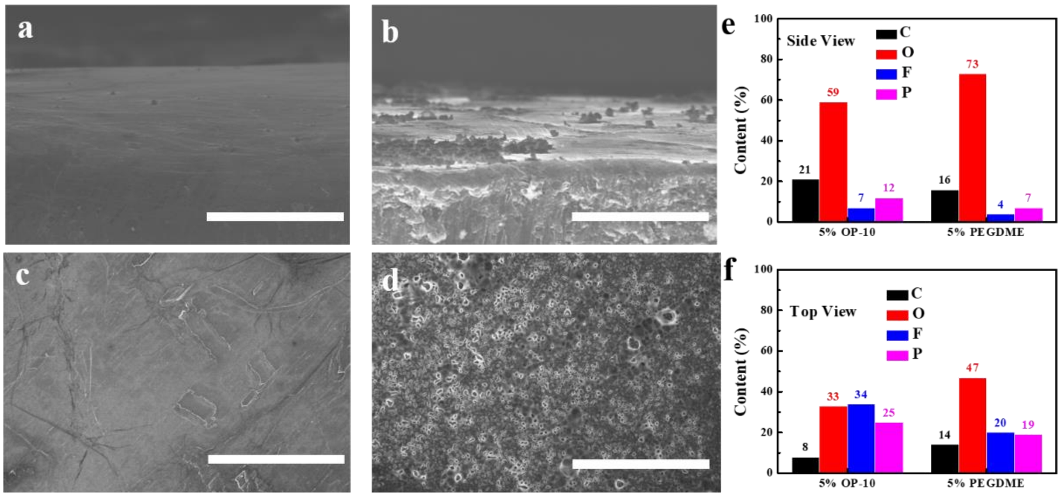

Supplementary Figure 2. Morphology and element characterizations of metal Li anode. SEM images of Li anode with OP-10 additives (**a, c**) or with PEGDME additives (**b, d**) after 3 cycles (**a, b**: side view; **c, d**: top view); Elements content of Li surface using various additives measured by EDX method after 3 cycles (**e, f**) (Scale bars: **a–d** 100 μm).

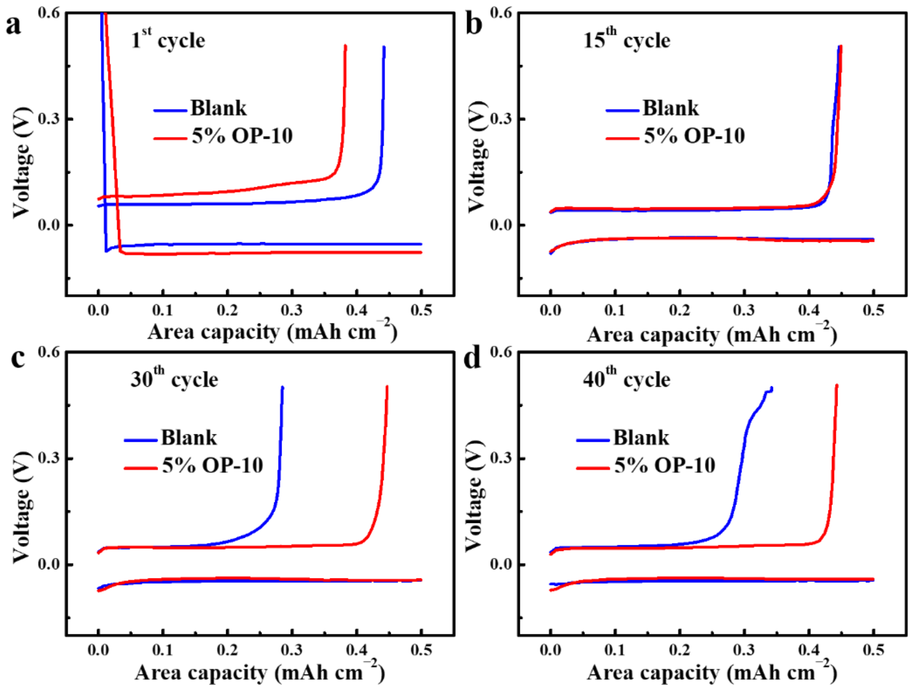

Supplementary Figure 3. Coulombic efficiency in different electrolyte systems. The voltage profiles of the 1st (**a**), 15th (**b**), 30th (**c**), and 40th (**d**) cycles in different electrolyte systems with a cycling capacity of 0.5 mAh cm^{-2} at a current density of 0.5 mA cm^{-2}.

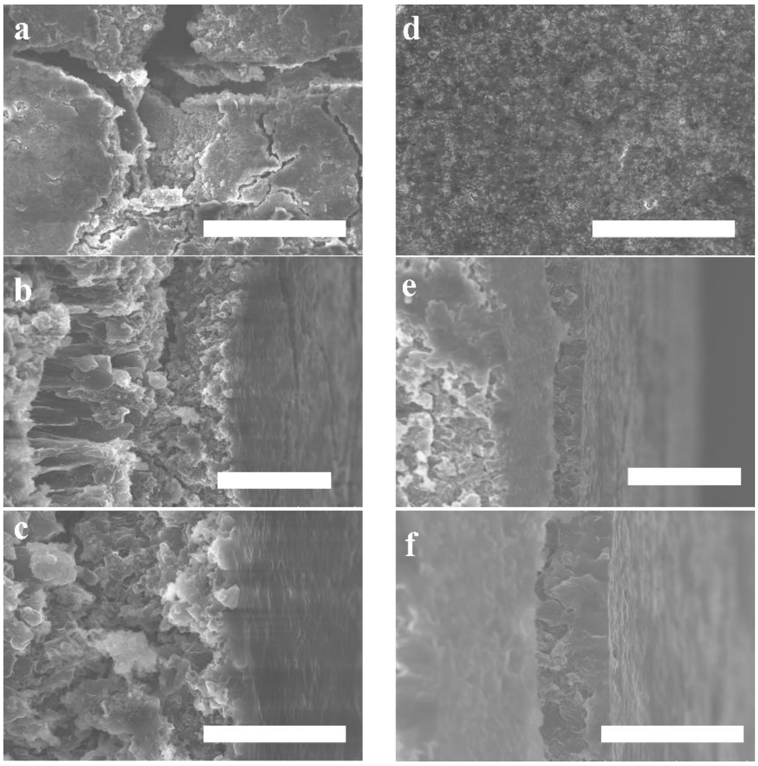

Supplementary Figure 4. Morphology characterizations of metal Li anode. SEM images of Li anode plated without OP-10 additives (**a, b, c**) or with OP-10 additives (**d, e, f**) after 50 cycles at a current density of 4 mA cm^{-2} with a fixed capacity of 1 mAh cm^{-2} (**a, d**: top view; **b, c, e, f**: side view. Scale bars: **a** 50 μm, **b** 20 μm, **c** 10 μm, **d** 50 μm, **e** 20 μm, **f** 10 μm).

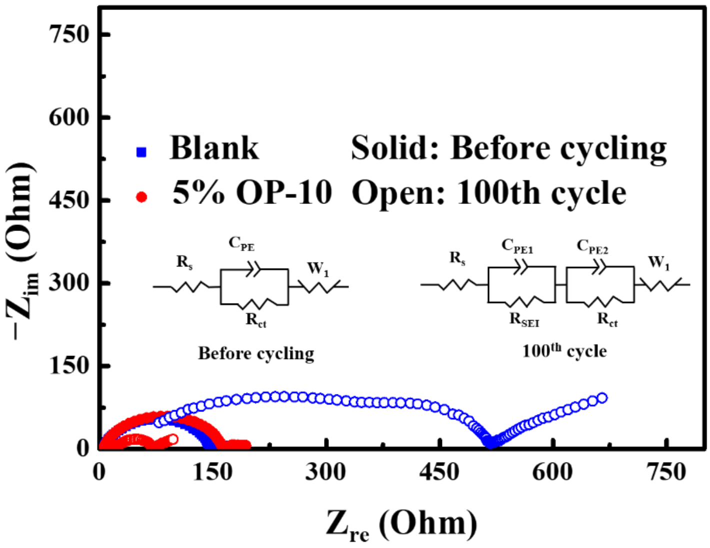

Supplementary Figure 5. Electrochemical impedance spectroscopy (EIS) results for blank and modified electrolytes before and after cycling at a current density of 2 mA cm^{-2} and a fixed capacity of 1 mA cm^{-2}. Inset figures corresponding to the equivalent circuits of the electrochemical impedance spectroscopy.

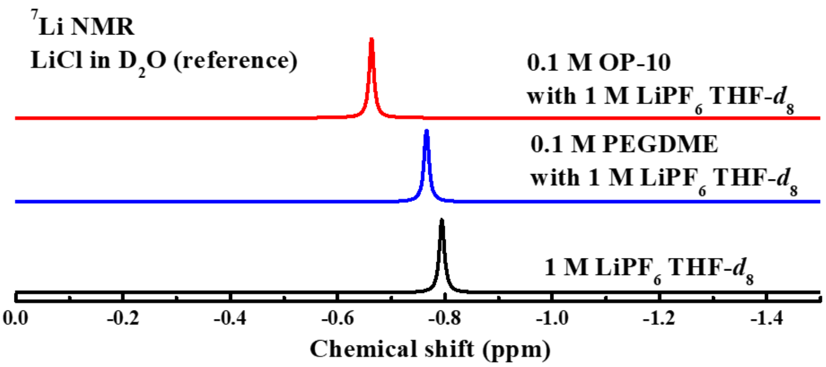

Supplementary Figure 6. ^{7}Li NMR spectra of $LiPF_6$ with different additives.

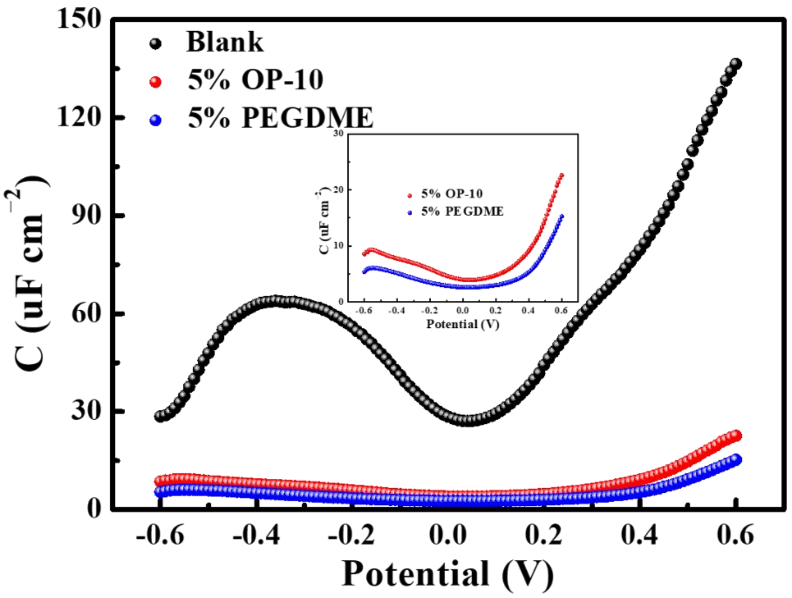

Supplementary Figure 7. Differential capacitance curves conducted at 298 K with or without OP-10 or PEGDME additives.

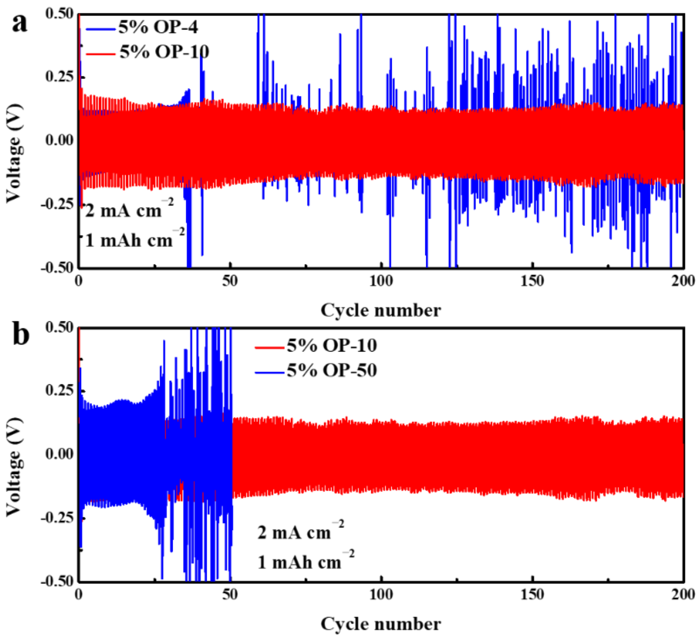

Supplementary Figure 8. Electrochemical performance with different kinds of OP additives. Cycling performance of Li|Li symmetric cells containing additives of OP-10 (red) versus OP-4 (**a**) and OP-50 (**b**) (blue).

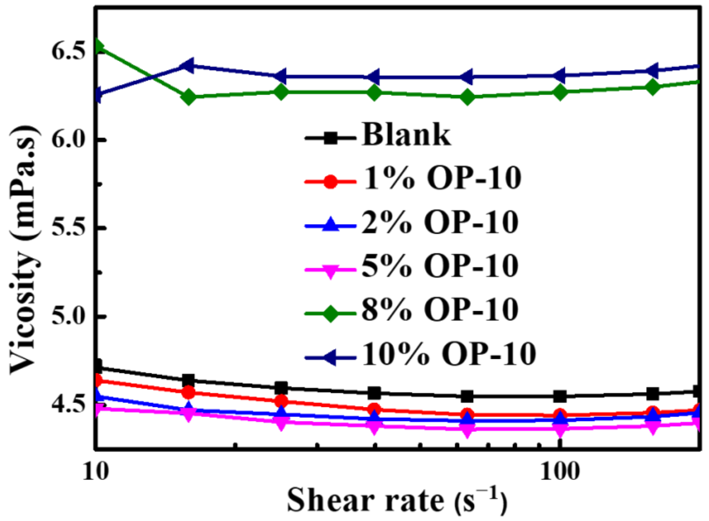

Supplementary Figure 9. Viscosities of OP-10 electrolyte additive with different concentrations.

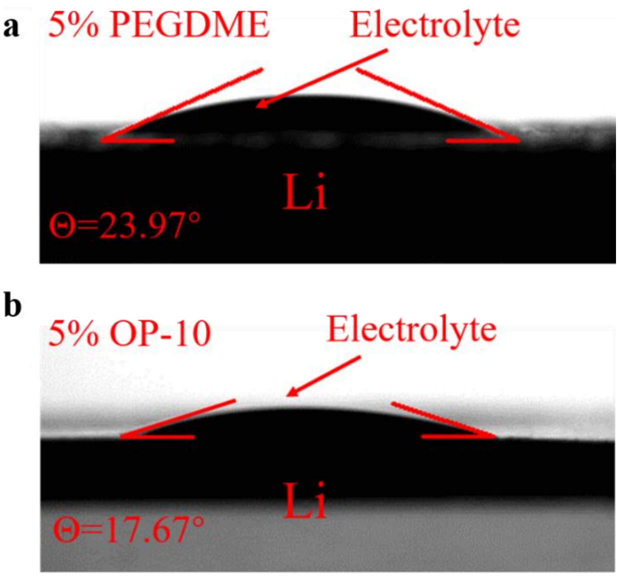

Supplementary Figure 10. Measurements of contact angles. Contact angles of the electrolyte with 5% PEGDME (**a**) and 5% OP-10 (**b**) additives on lithium anode.

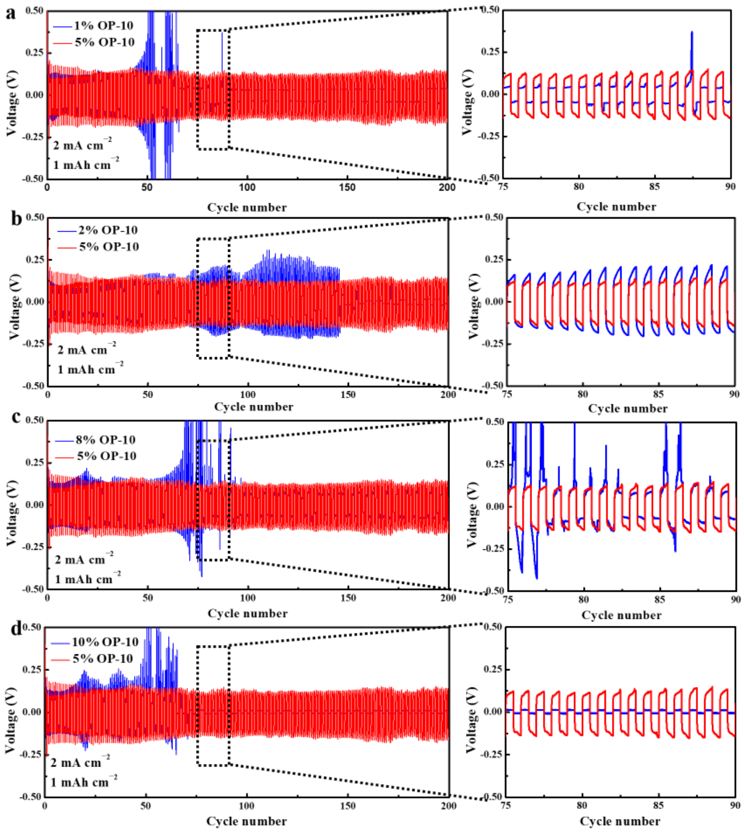

Supplementary Figure 11. Electrochemical performance of Li|Li symmetric cells. Cycling performance of Li|Li cells using 1% (**a**), 2% (**b**), 8% (**c**) and 10% (**d**) OP-10 additive.

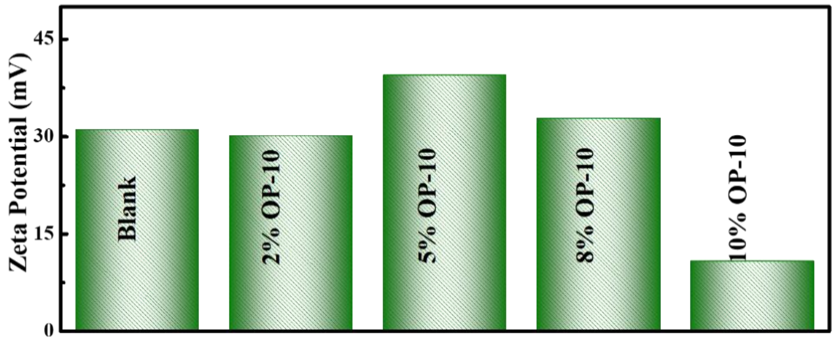

Supplementary Figure 12. Zeta potential of lithium without or with OP-10 additives at different concentration.

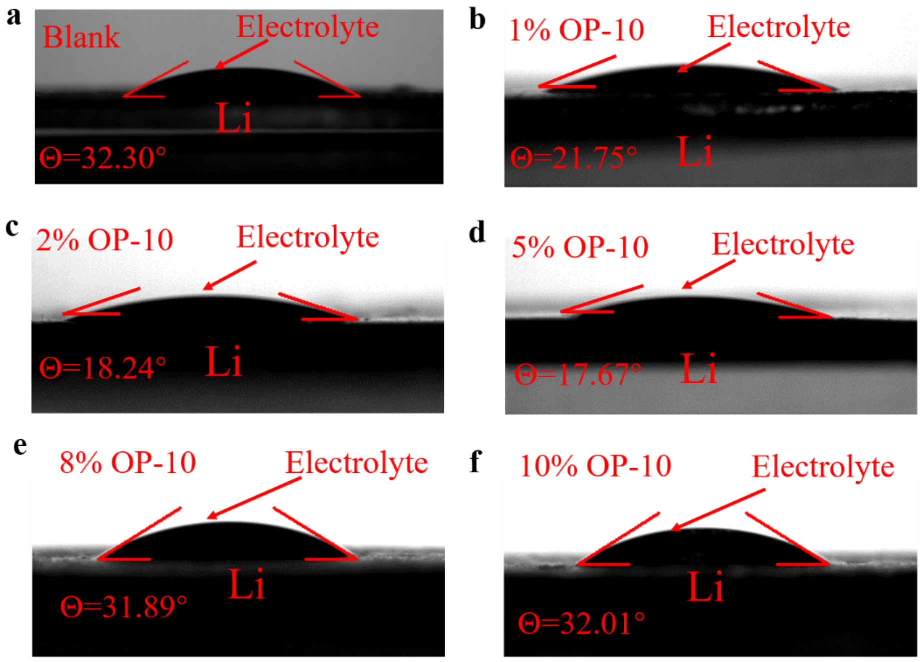

Supplementary Figure 13. Measurements of contact angles. Contact angles of electrolytes with 1% (**a**), 2% (**b**), 8% (**c**) and 10% (**d**) OP-10 additive on lithium anode.

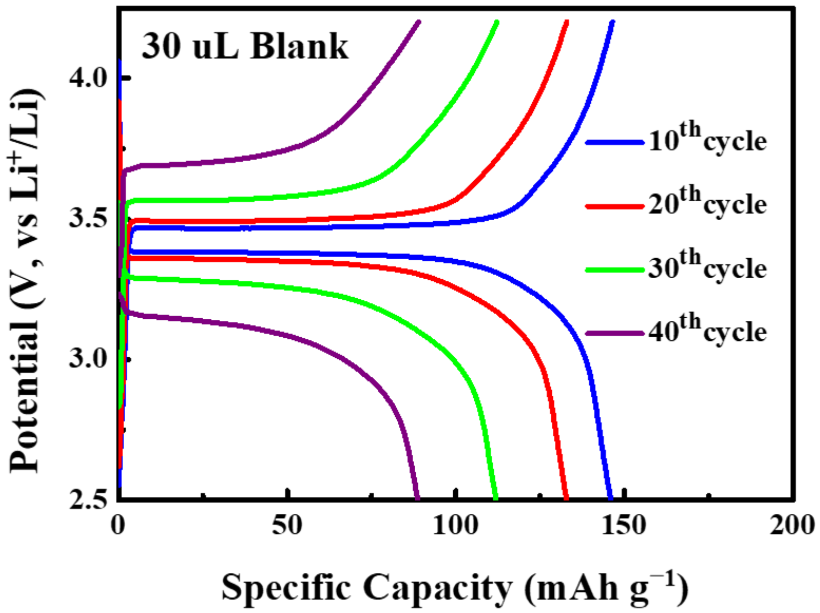

Supplementary Figure 14. Charge-discharge profiles of the as-assembled Li|$LiFePO_4$ batteries with untreated electrolyte corresponding to Fig. 6a.

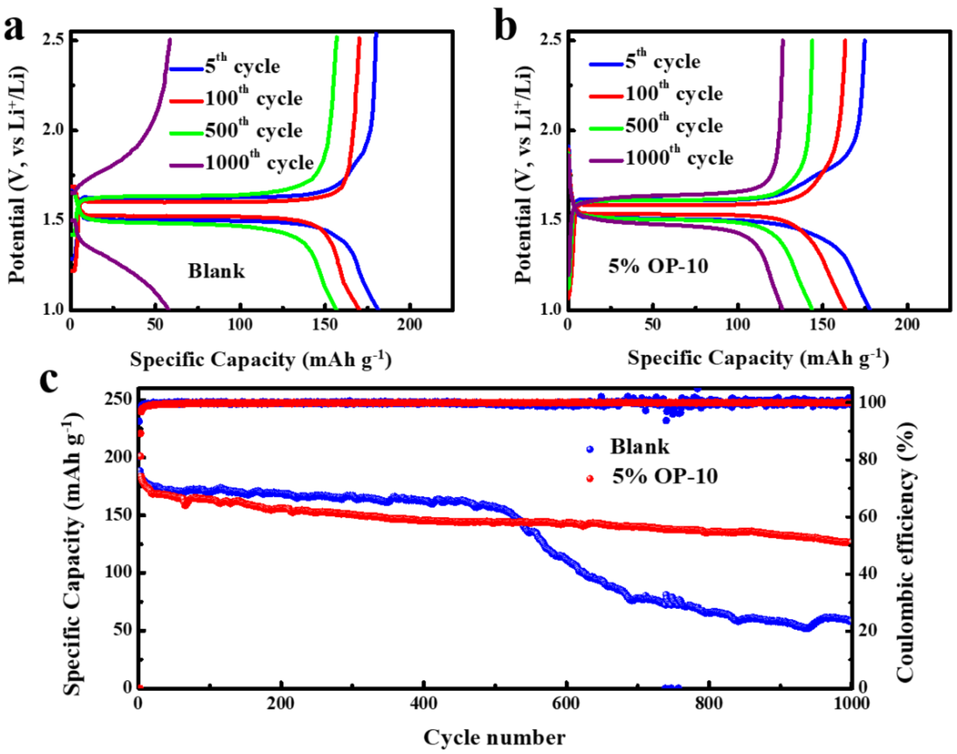

Supplementary Figure 15. Electrochemical performances of Li|$Li_4Ti_5O_{12}$ batteries with untreated electrolyte (blue) or with OP-10 additives (red). Charge-discharge profiles of $Li_4Ti_5O_{12}$ batteries with untreated electrolyte (**a**) or with OP-10 additives (**b**) at 5 C; (**c**) Long-term cycling stability of Li| $Li_4Ti_5O_{12}$ batteries with untreated electrolyte or with OP-10 additives at a current density of 5 C. (1 C = 175 mA g^{-1}).

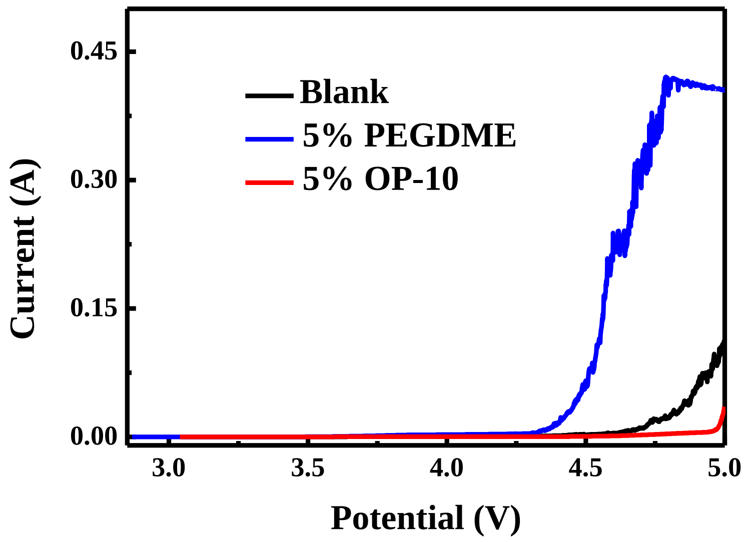

Supplementary Figure 16. Linear sweep voltammetry (LSV) curves of Li|Cu cells assembled with or without OP-10/PEGDME at a scan rate of 10 mV s^{-1}.

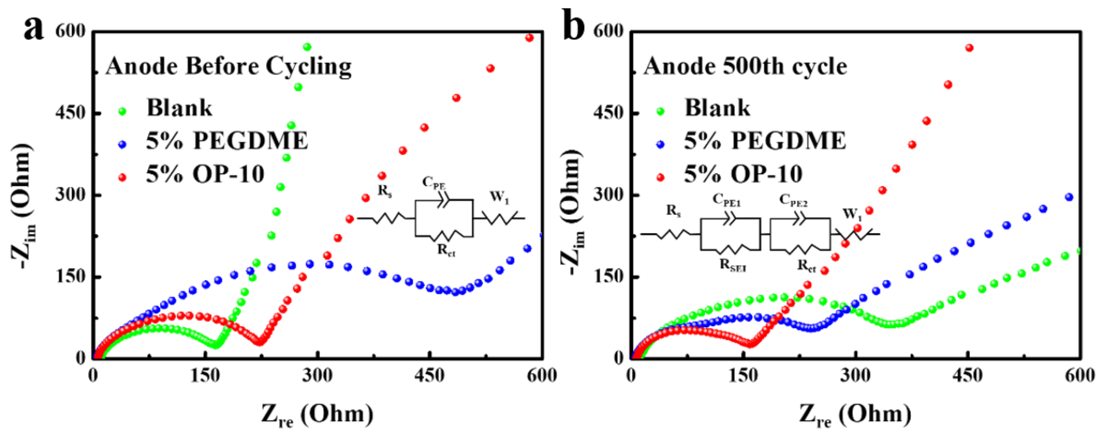

Supplementary Figure 17. EIS measurements of Li anode in Li|$LiFePO_4$ battery. Nyquist plots before cycling (**a**) and after 500 cycles (**b**) using electrolyte with or without additives. Inset figures are the equivalent circuits of the electrode impedance spectra.

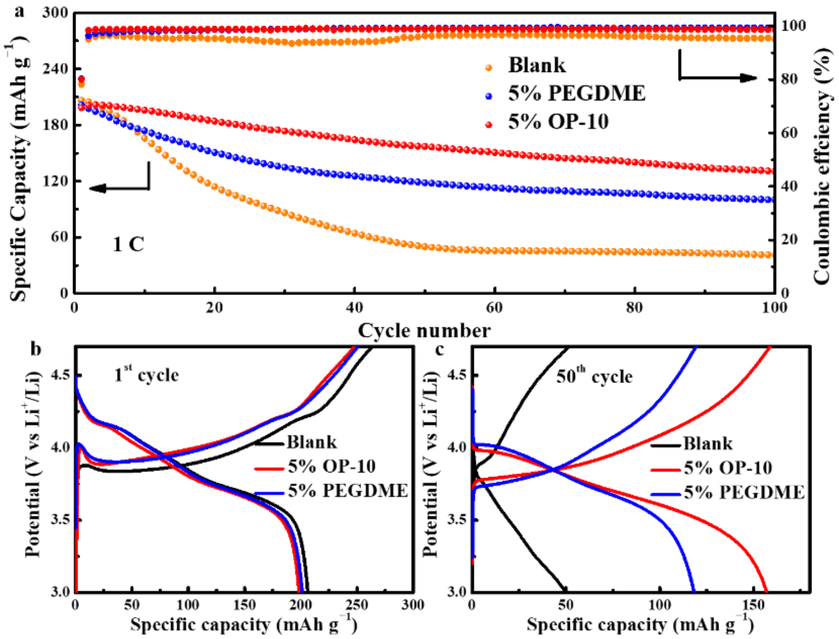

Supplementary Figure 18. Electrochemical performance of Li|$LiNi_{0.8}Co_{0.1}Mn_{0.1}O_2$ batteries in different electrolyte systems. Cycling performance of Li|$LiNi_{0.8}Co_{0.1}Mn_{0.1}O_2$ full cells with or without OP-10/PEGDME additives at a current density of 1 C (1 C = 280 mAh g^{-1}) (**a**); Charge-discharge profiles of Li|$LiNi_{0.8}Co_{0.1}Mn_{0.1}O_2$ full cell in different electrolyte systems (**b–c**).

Supplementary Table 1. The comparisons of the bare Li foil between our work and previous reports

Electrolyte additives	Current density (mA cm^{-2})	Areal capacity (mAh cm^{-2})	Overpotential (mV)	Cycle number	Ref.
1.0 M (Pyr1(12) FSI	0.5	2.0	30	～110	1
0.05 M $LiPF_6$ + dual-salt	1.0	0.5	～200	210	2
8 wt% $AlCl_3$	0.5	1.0	50	～235	3
8% polydimethylsiloxane	0.5	1.5	100	300	4
20 mM Boric acid	0.25	0.5	<80	215	5
0.15 M LiDFOB	1	1	～100	300	6
60 mM InF_3	1	1	100	200	7
1.2 mM CTAC	1	0.5	～69	300	8
5% Lithium Nitrate	0.5	0.5	52	150	9
5% OP-10	1	0.5	～100	400	**This Work**
	2	1	～130.2	200	
	4	1	～188.5	160	

Supplementary Table 2. The corresponding fitting results of the Supplementary Figure 5

Fitting results			
		Blank	5% OP-10
Before cycle	R_{ct} (ohm)	140.5	156.2
100th cycle	R_{SEI} (ohm)	345.4	32.36
	R_{ct} (ohm)	143.6	28.88

Supplementary Table 3. Elements content of Li surface measured by EDX method with or without OP-10 additives

Sample	C (%)	O (%)	F (%)	P (%)
Blank	12	49	20	19
5% OP-10	8	33	34	25

Supplementary Table 4. The corresponding fitting results of the Supplementary Figure 17

Fitting results				
		Blank	5% PEGDME	5% OP-10
Before cycle	R_{ct} (ohm)	154.2	466.6	210.1
500th cycle	R_{SEI} (ohm)	302.3	76.27	68.5
	R_{ct} (ohm)	23.96	138.7	76.32

Supplementary References

1 Yoo, D., Kim, K. & Choi, J. The synergistic effect of cation and anion of an ionic liquid additive for lithium metal anodes. *Adv. Energy Mater*. **8**, 1702744 (2018).

2 Zheng, J. et al. Electrolyte additive enabled fast charging and stable cycling lithium metal batteries. *Nat. Energy* **2**, 17012 (2017).

3 Ye, H.et al. Synergism of Al-containing solid electrolyte interphase layer and Al-based colloidal particles for stable lithium anode. *Nano Energy* **36**, 411–417 (2017).

4 Meng, J., Chu, F., Hu, J. & Li, C. Liquid polydimethylsiloxane grafting to enable dendrite-free li plating for highly reversible li-metal batteries. *Adv. Funct. Mater*. **29**, 1902220 (2019).

5 Huang, Z.et al. Protecting the Li-metal anode in a Li-O_2 battery by using boric acid as an SEI-forming additive. *Adv. Mater*. **30**, 1803270 (2018).

6 Yu, L.et al. A localized high-concentration electrolyte with optimized solvents and lithium difluoro(oxalate)borate additive for stable lithium metal batteries. *ACS Energy Lett*. **3**, 2059–2067 (2018).

7 Pang, Q., Liang, X., Kochetkov, I., Hartmann, P. & Nazar, L. Stabilizing lithium plating by a biphasic surface layer formed in situ. *Angew. Chem. Int. Ed*. **57**, 9795–9798 (2018).

8 Dai, H., Xi, K., Liu, X., Lai, C. & Zhang, S. Q. Cationic surfactant-based electrolyte additives for uniform lithium deposition via lithiophobic repulsion mechanisms. *J. Am. Chem. Soc*. **140**, 17515–17521 (2018).

9 Tan, S.-J. et al. Nitriding-interface-regulated lithium plating enables flame-retardant electrolytes for high-voltage lithium metal batteries. *Angew. Chem. Int. Ed*. **58**, 7802–7807 (2019).
